# Advancing systemic therapy for biliary tract cancer: current strategies and emerging paradigms

**DOI:** 10.3389/fonc.2026.1769447

**Published:** 2026-02-11

**Authors:** Khalil Choucair, Shadi Chamseddine, Asfar Azmi, Philip A. Philip

**Affiliations:** 1 Barbara Ann Karmanos Cancer Institute, Wayne State University School of Medicine, Detroit, MI, United States; 2 Department of Internal Medicine, Wayne State University, Detroit, MI, United States; 3 Henry Ford Hospital, Detroit, MI, United States; 4 Department of Oncology, Wayne State University School of Medicine, Detroit, MI, United States; 5 Department of Pharmacology, Wayne State University School of Medicine, Detroit, MI, United States

**Keywords:** biliary tract cancer, immunotherapy, molecular profiling, systemic chemotherapy, targeted therapy

## Abstract

Most patients diagnosed with biliary tract cancer (BTC) present with advanced, often unresectable disease. Additionally, a significant proportion of those who undergo curative intent resection experience disease recurrence. The aggressive nature and poor prognosis of BTC underscore the urgent need for effective and safe systemic therapies. For many years, progress in the therapy of BTC was limited. However, advances in our understanding of the molecular biology of BTC created a paradigm shift in its treatment. Novel therapies, particularly personalized approaches based on genomic profiling and use of targeted treatments, have significantly transformed the management algorithm for advanced BTC. These advances represented a major step forward in improving outcomes for patients with this very challenging malignancy.

## Highlights

- Advances in BTC therapy include a shift from cytotoxic chemotherapy alone to biomarker-driven targeted therapies and chemo-immunotherapy combinations.
- Molecular profiling enables personalized BTC treatment by identifying actionable alterations such as FGFR2 fusions, IDH1/2 mutations, and HER2 amplification/overexpression.
- FGFR inhibitors (pemigatinib, futibatinib) and IDH1 inhibitors (ivosidenib) improve outcomes in selected BTC subtypes and have reshaped second-line management.
- The TOPAZ-1 and KEYNOTE-966 trials established gemcitabine/cisplatin plus durvalumab or pembrolizumab as new first-line standards of care for advanced BTC, with consistent overall survival benefits versus chemotherapy alone.
- Recently approved HER2-directed antibodies (zanidatamab) and active HER2 combinations (tucatinib plus trastuzumab), along with next-generation FGFR inhibitors and chemo-IO combinations, further expand the BTC treatment landscape and illustrate the rapid evolution of systemic therapy.

## 1 Introduction

**Figure 1:**
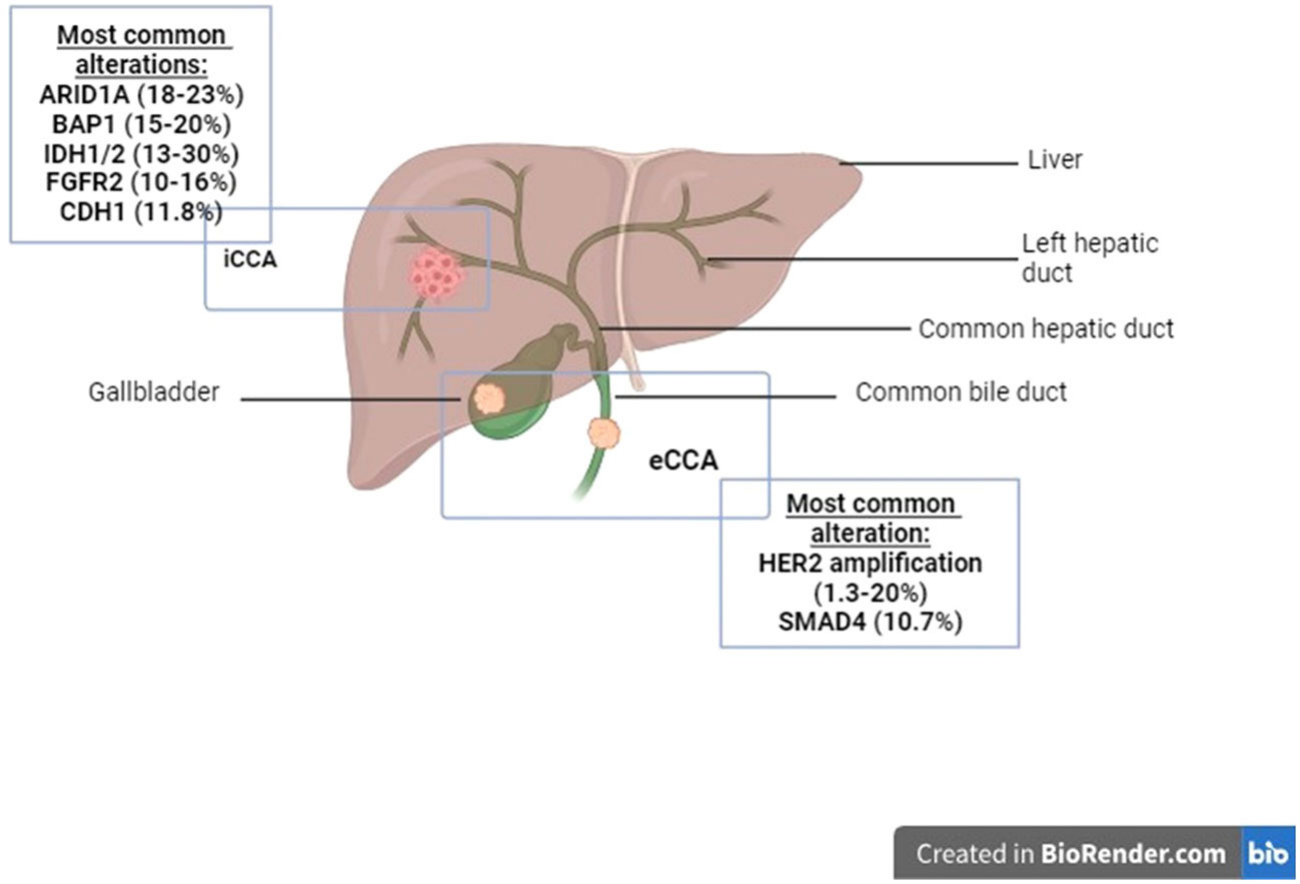
Anatomy of the biliary tree and biliary tract cancers (BTC): BTCs include gallbladder cancer, intrahepatic cholangiocarcinoma (iCCA) and extrahepatic cholangiocarcinoma (eCCA). Frequencies of the molecular alterations are variable within different anatomic locations of the biliary tree, and are listed above.

Biliary tract cancers (BTCs) encompass a heterogeneous group of malignancies, including gallbladder cancer (GBC), intrahepatic cholangiocarcinoma (iCCA), and extrahepatic cholangiocarcinoma (eCCA) (1) (**Figure 1**). These cancers are frequently incurable and associated with poor prognosis, largely due to late-stage diagnoses and presence of systemic disease. Symptoms are often vague and nonspecific, contributing to the delayed detection. Consequently, the majority of patients present with unresectable tumors, and even among those who undergo successful curative intent surgical resection, up to two-thirds experience disease recurrence (2). While surgical resection with or without adjuvant therapy can provide a potential cure, this is achievable in only a minority of cases, underscoring the critical need for effective systemic therapies to prolong survival and palliate tumor-related symptoms.

The incidence of BTC varies significantly across the globe, with geographic disparities reflecting some unique regional risk factors. Higher incidence rates are observed in Asian countries such as northern India, Pakistan, Japan, and Korea, as well as in South American nations like Bolivia, Ecuador, and Chile, which report the highest mortality rates worldwide (3–5). These regions share common risk factors, including prevalence of gallstones and chronic salmonella infections (6–8), alongside systemic challenges to timely diagnosis (9). In contrast, BTC is considered a low-incidence malignancy in Western countries. For example, in the United States, recent data from the Surveillance, Epidemiology, and End Results (SEER) database estimate an incidence of 1 to 2 cases per 100,000 population (10).

Despite its low incidence, BTC is an aggressive group of malignancies, with an estimated 5-year overall survival (OS) rate of just 8-10% and a median OS (mOS) of approximately 1 year for patients with advanced disease (11, 12). This review explores the critical role and challenges in developing effective systemic therapies while highlighting the rapidly evolving promise of molecularly targeted therapies in transforming the treatment landscape for BTC.

## 2 Systemic cytotoxic therapies

Systemic therapy with conventional cytotoxic agents remains palliative, offering limited survival benefits (**Table 1**). This section examines the available first-line and subsequent cytotoxic chemotherapy options for managing advanced GBC.

**Table 1:** Summary of systemic and immune-based therapies.

Trial name^(ref.)^	Phase	Treatment arms	N	L	ORR (%)	PFS (months)	OS (months)	NCT
SYSTEMIC CYTOTOXIC CHEMOTHERAPY								
ABC-02	III	Gem/Cis (*vs.* Gem)	204 (*vs.*206)	1	26.1 (*vs.* 15.5)	8.0 (*vs.* 5.0)	11.7 (*vs.* 8.1)	00262769
BT-22	II	Gem/cis (*vs.* Gem)	42 (*vs.* 42)	1	19.5 (*vs.* 11.9)	5.8 (*vs.* 3.7)	11.2 (*vs.* 7.7)	–
CTRI/2010/091/001406	III	mGemox (*vs.* Gem/cis)	119 (*vs.*124)	1	22.6 (*vs.* 23.6)	6 (*vs.* 4.5; p=0.123)	9 (*vs.* 8; p=0.152)	CRTI/2010-091-001406
SWOG1815	III	GAP (*vs.* Gem/Cis)	299 (*vs.* 153)	1	31 (*vs.* 21; p=0.03)	7.5 (*vs.* 6.3; p=0.32)	14 (*vs.* 13.6; p=0.41)	03768414
NIFTY	II-b	5-FU/LV/LI (*vs.* 5-FU/LV)	88 (*vs.* 86)	2	N/A	7.1 (*vs.* 1.4; p=0.0019)	N/A	03524508
NALIRICC-AIO- HEP-0116	II	5-FU/LV/LI (*vs.* 5-FU/LV)	49 (*vs.* 51)	2	14.3 (*vs.* 3.9)	2.76 (*vs.* 2.3)	6.9 (*vs.* 8.21)	–
SYSTEMIC IMMUNE-BASED THERAPIES								
KN-028	I-b	Pembrolizumab	24	2+	13	1.8	6.2	02054806
KN-158	II	Pembrolizumab	104	2+	5.8	2.0	7.4	02628067
NCT02829918	II	Nivolumab	54	2-3	22	5.7	14.2	02829918
CA209-538	II	Nivolumab+ ipilimumab	39	2+	23	2.9	5.7	02923934
NCT03311789	II	Gem/Cis + nivolumab	32	1-2+	55.6 (L1:61.9;2+:33)	6.1	8.5	03311789
TOPAZ-1	III	Gem/Cis/durvalumab (*vs.* Gem/Cis/placebo)	341 (*vs.* 344)	1	26.7 (*vs.* 18.7)	HR: 0.80 (p=0.021)	HR: 0.75 (p=0.01)	03875235
KN-966	III	Gem/Cis/Pembrolizumab (*vs.* Gem/Cis/Placebo)	533 (*vs.* 536)	1	29 (*vs.* 29)	6.5 (*vs.* 5.6; p=0.023)	12.7 (*vs.* 10.9; p=0.0054)	04003636
IMbrave 151	II	Bevacizumab/Gem/Cis/Atezolizumab (*vs.* Gem/Cis/Atezolizumab)	79 (*vs.* 83)	1	24 (*vs.* 25)	8.4 (*vs.* 7.9)	NR	04677504

NCT#, National Clinical Trial registration number; Line (L), 1, 2, 3; 2+, 2 and beyond; ORR, Objective response rate; PFS, progression-free survival; OS, overall survival; HR, Hazard Ratio; Gem, Gemcitabine; Cis, Cisplatin; GAP, Gemcitabine/Cisplatin/Nab-paclitaxel; LV, Leucovorin; LI, Liposomal Irinotecan; CR, Complete response; N/A, not applicable/not reported; NR, not reached; ref, reference.

### 2.1 Front-line therapies

#### 2.1.1 Single-agent therapies

Single-agent cytotoxic therapies are now considered historical and are primarily discussed here to contextualize the development of modern combination regimens. Early studies comparing single-agent chemotherapies, such as fluoropyrimidines and gemcitabine, to best supportive care established their role as foundational treatments for combination regimens. For example, the oral fluoropyrimidine agent S1 achieved a 45% objective response rate (ORR) and a mOS of 8.1 months in a phase II study of 20 patients (13). Similarly, 5-fluorouracil (5-FU) with leucovorin yielded a 5% partial response (PR) and a 55% disease-control rate (DCR) in another 20-patient study (14). Gemcitabine monotherapy demonstrated response rates of 7–36% across multiple studies, with one trial reporting a 35.7% PR, 78.6% DCR, and a median time to progression (mTTP) of 6.4 months (95% CI: 5.8-7.1) and mOS of 17.1 months (95% CI: 15.8-18.5) (15–17). Geographic differences in efficacy have been noted, such as lower response rates in Japan (ORR: 8.8%) compared to other regions (15, 18). Other single agents, including irinotecan (ORR: 8.7%; median progression-free survival (mPFS): 2.7 months; mOS: 7.0 months; n=23) (19) and capecitabine (DCR: 19%; mOS: 8.1 months; n=26) (20), have also been evaluated, though with limited success, highlighting the need for more effective therapeutic strategies.

### 2.2 Doublet therapy

#### 2.2.1 Gemcitabine and cisplatin

Non-randomized clinical studies demonstrated ORRs ranging from 22.7% to 36.7% (21–23). The ABC-01 trial was the first randomized study to evaluate cisplatin and gemcitabine in advanced/metastatic BTC, leading to the phase III ABC-02 trial, which compared gemcitabine monotherapy to the combination therapy (11). ABC-02 showed a significant survival benefit for the doublet over single agent gemcitabine, with mPFS of 8.0 months versus 5.0 months and mOS of 11.7 months versus 8.1 months. Subset analysis revealed that patients with GBC had improved outcomes with the doublet, showing higher PR rates (37.7% *vs*. 21.4%), DCR (85.2% *vs*. 76.8%), and lower rates of disease progression (14.8% *vs*. 23.2%). The BT22 trial, a phase II study, supported these findings, reporting a tendency toward longer survival for patients receiving the doublet compared to gemcitabine alone (mOS: 9.1 months *vs*. 6.7 months; p=0.675) (24). Gemcitabine and cisplatin became the standard first-line treatment for metastatic or unresectable BTC. It is currently the cytotoxic platform of choice to which other agents are added.

#### 2.2.2 Other doublet regimens

Gemcitabine combined with oxaliplatin (GEMOX) has shown varied efficacy across studies (25–28). In 33 advanced GBC patients, GEMOX demonstrated an ORR of 36%, stable disease in 88%, mTTP of 5.3 months, and mOS of 6.8 months (25). A smaller study (n=10) reported a 40% PR rate, DCR of 70%, and mOS of 11.1 months (26). However, a 3-weekly regimen in 48 BTC patients showed lower ORR (21.2%) and DCR (56.6%) with mOS of 7.5 months (27). Similarly, an international phase II trial reported an ORR of only 4.3% and mPFS of 2.5 months (28).

Gemcitabine combined with capecitabine yielded comparable ORRs to GEMOX, with PR rates of 33%, DCR of 64–75%, mPFS of 4.4 months, and mOS of 7.7–16 months (29, 30). In combination with pemetrexed, gemcitabine showed a CR rate of 6.3% and ORR of 12.5% (31). Historically, a phase III trial comparing modified GEMOX to gemcitabine/cisplatin reported similar ORR, PFS and OS between the two doublets. However, subsequent pooled analyses and real-world data have generally favored cisplatin/gemcitabine, particularly once immunotherapy (durvalumab or pembrolizumab) was added to this backbone. As a result, GEMOX is now typically reserved for cisplatin-ineligible or frail patients rather than considered an equivalent first-line standard (32).

Non-gemcitabine doublets include capecitabine/cisplatin, which showed a PR rate of 53.3% in one study but lower ORRs of 14.2–32% in others (33–35). Cisplatin with 5-FU demonstrated PR rates of 46.7% and 36% in Japanese and French studies, respectively, but short survival durations (mOS: 4.9 months) in the Japanese cohort (36, 37). A combination of S1 and oxaliplatin achieved a PR rate of 40% in a small cohort (n=10) (38). Finally, oxaliplatin with capecitabine reported an ORR of 30%, DCR of 63%, mTTP of 4.7 months, and mOS of 8.0 months in a German prospective multicenter phase II study (39).

#### 2.2.3 Triplet and quadruple regimens

Building on the success of doublet therapies, triplet combinations were explored for advanced BTC. A regimen of 5-FU, gemcitabine, and cisplatin demonstrated an ORR of 40% with one CR in 15 patients, but with significant grade 4 neutropenia (71.4%) (40). A triplet of gemcitabine, 5-FU, and oxaliplatin showed a PR rate of 23%, DCR of 69%, mTTP of 5.7 months, and mOS of 9.9 months in 35 patients with advanced GBC (41). Another triplet of gemcitabine, leucovorin, and 5-FU reported a PR rate of 21.4%, mPFS of 5.2 months, and mOS of 7.2 months in 14 patients (42).

##### 2.2.3.1 The SWOG 1815 trial and GAP in first-line treatment of advanced BTC

In a phase II trial, the addition of albumin-bound paclitaxel to gemcitabine and cisplatin (GAP) demonstrated promising results in 60 patients, with an ORR of 45%, mPFS of 11.8 months, and mOS of 19.2 months (43). In the phase III SWOG 1815 trial, the largest phase III trial in advanced BTC, GAP was compared to gemcitabine/cisplatin in 441 patients. GAP achieved an mOS of 14 months compared to 12.7 months for gemcitabine/cisplatin (HR 0.93, p=0.58), with no significant differences in ORR (34% *vs*. 25%; p=0.11) or mPFS (8.2 *vs*. 6.4 months; HR 0.92, p=0.47) (44). GAP was associated with higher grade 3+ hematologic toxicity (60% *vs*. 45%; p=0.003) but similar treatment discontinuation rates (24% *vs*. 19%; p=0.26). Subgroup analysis suggested better mOS for GAP in patients with locally advanced disease (19.2 *vs*. 13.7 months; HR 0.67, p=0.09) and GBC patients (17.0 *vs*. 9.3 months; HR 0.74, p=0.33). While GAP did not show significant mOS improvement overall, these findings emphasize differences in tumor biology and disease stage, discussed further in the targeted therapies section. The most updated results from this trial, published in December 2024, further confirmed those results, with no difference in OS, and increased toxicity associated with GAP (44). The lack of a significant survival benefit with GAP may reflect the biological heterogeneity of BTC and a potential ceiling effect of intensified cytotoxic therapy in an unselected population. These findings emphasize the need for biomarker-enriched or biology-driven trial designs rather than further escalation of chemotherapy intensity alone.

##### 2.2.3.2 5-FU-based non-gemcitabine triple and quadruple therapies

Several 5-FU-based regimens without gemcitabine have been evaluated. A combination of 5-FU with high-dose levofolinic acid and oral hydroxyurea showed a PR rate of 30%, DCR of 57%, and mOS of 8 months (45). A German phase II study of irinotecan, folinic acid, and 5-FU reported lower PR rates (15%), DCR of 31%, mPFS of 5.3 months, and mOS of 9.1 months (46). FOLFIRINOX, traditionally used in the second-line setting, was evaluated as a first-line option in a phase II study of 35 patients, achieving an mPFS of 7.4 months, mOS of 14.7 months, and ORR of 31.4% with a DCR of 74.3%. However, the study did not meet its primary endpoint, and no phase III trial was pursued (47). Importantly, higher-level evidence from the randomized phase II/III PRODIGE 38/AMEBICA trial demonstrated no improvement in survival outcomes with modified FOLFIRINOX compared with standard gemcitabine/cisplatin, while being associated with increased toxicity (48). Collectively, these data do not support the use of FOLFIRINOX as a first-line regimen in unselected BTC patients.

### 2.3 Second line and beyond

Until 2019, there was no consensus on the benefit of second-line cytotoxic chemotherapy, with most evidence coming from small retrospective studies (reviewed in (49)). Systematic reviews and meta-analyses of these studies reported mOS of 6.5–7.2 months, PFS of 2.6–3.2 months, and an ORR of 7.7% (50, 51).

#### 2.3.1 Single-agent chemotherapy in the second line setting

Fluoropyrimidines are the only monotherapies evaluated for unresectable and advanced GBC in the second-line setting. S-1 monotherapy (80 mg/m² for 28 days followed by 14 days of rest) was first studied in 2009, showing a response rate of 18.8%, mPFS of 5.5 months, mOS of 8.0 months, and a 1-year survival rate of 38.2% in 16 second-line patients (52). A subsequent multicenter phase II study in 22 gemcitabine-refractory BTC patients reported an ORR of 22.7%, DCR of 50%, mOS of 13.5 months, and mTTP of 5.4 months (53). However, a more recent study in 14 GBC patients showed lower efficacy, with an ORR of 7.1%, mPFS of 1.4 months, and mOS of 4.7 months (54).

#### 2.3.2 Combination regimens in the second line setting

Given the modest efficacy of single-agent fluoropyrimidine, fluoropyrimidine-based combination regimens were explored for advanced BTC.

##### 2.3.2.1 FOLFOX

FOLFOX showed an ORR of 24.2%, DCR of 59.1%, mTTP of 3.9 months, and mOS of 7.6 months in a prospective case series (55). Level-1 evidence emerged in 2021 through the ABC-06 trial, a phase III randomized study of 166 patients comparing active symptom control (ASC) alone to ASC plus FOLFOX (oxaliplatin, folinic acid, bolus fluorouracil, and continuous fluorouracil infusion) (56). FOLFOX+ASC significantly improved mOS (6.2 *vs*. 5.3 months; HR: 0.69, P = 0.031), with 6- and 12-month OS rates of 50.6% and 25.9%, respectively, compared to 35.5% and 11.4% with ASC alone (56). This trial provided the first robust evidence supporting FOLFOX as a second-line treatment for advanced BTC.

#### 2.3.3 5-FU and liposomal irinotecan in second-line BTC treatment

A phase IIb trial conducted in South Korea evaluated liposomal irinotecan plus fluorouracil/leucovorin versus fluorouracil/leucovorin alone in 174 patients with metastatic BTC who had progressed on gemcitabine and cisplatin. The experimental arm demonstrated a significantly longer PFS in the initial analysis (7.1 *vs*. 1.4 months; HR 0.56, p=0.0019) (57). However, subsequent analyses incorporating blinded independent central radiologic review reported a more modest PFS benefit, and no consistent overall survival advantage was observed. These findings raise uncertainty regarding the magnitude of benefit of liposomal irinotecan–based regimens in the second-line setting. Notably, the phase II NALIRICC-AIO-HEP-0116 trial conducted in Germany failed to confirm these results (58). In that study of 100 patients, liposomal irinotecan plus fluorouracil/leucovorin was associated with an mOS of 6.9 months and an mPFS of 2.76 months, compared with 8.21 months and 2.3 months, respectively, for fluorouracil/leucovorin alone, while also demonstrating higher rates of grade ≥3 toxicity (70.8% *vs*. 50%). Collectively, the available data suggest that the role of liposomal irinotecan plus fluorouracil/leucovorin in BTC remains uncertain, particularly in the absence of direct comparative data against FOLFOX, which remains the most commonly used second-line chemotherapy backbone.

#### 2.3.4 Other regimens in the second-line setting

**Figure 2:**
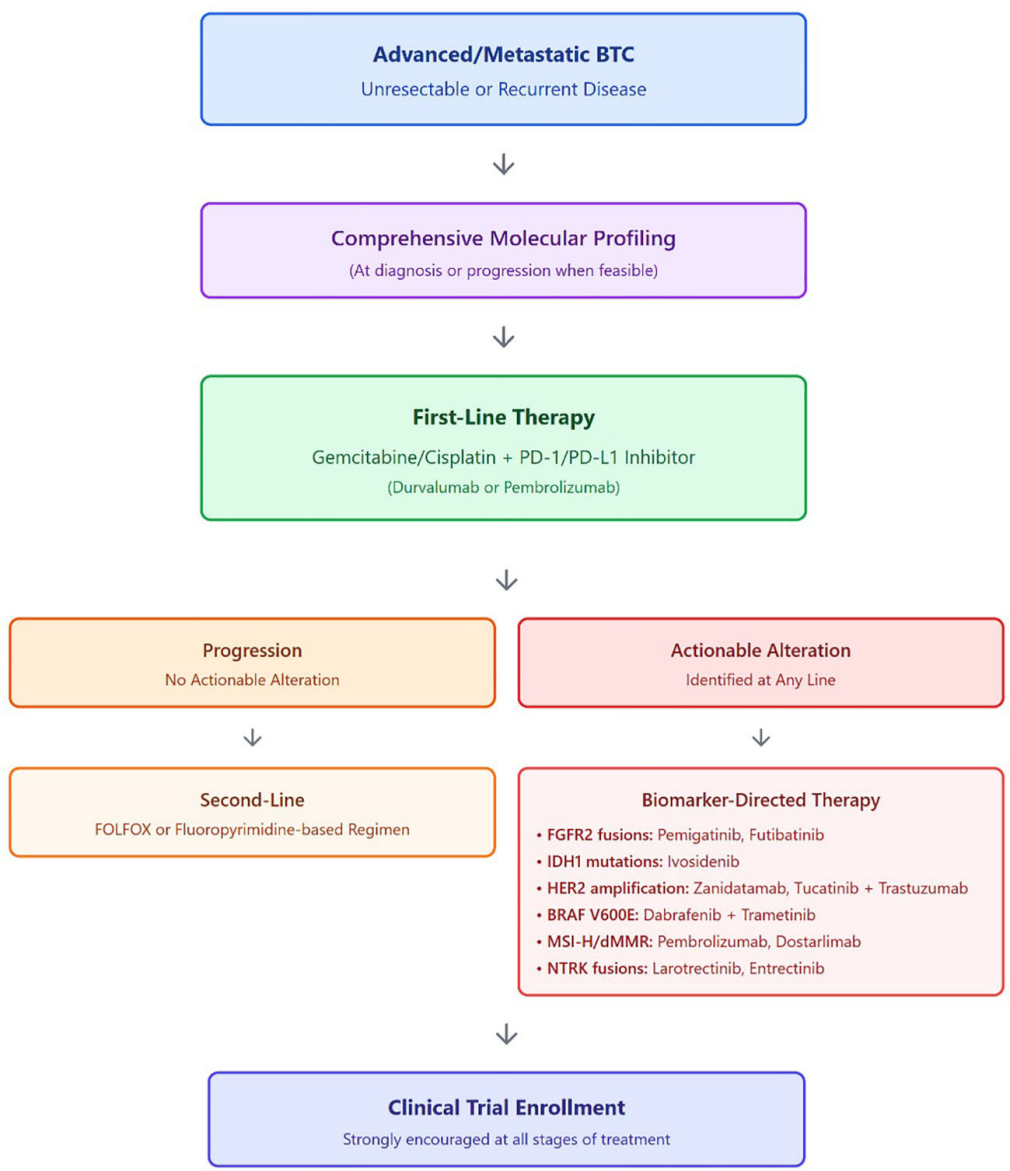
Contemporary systemic treatment framework for advanced biliary tract cancer: Schematic overview illustrating commonly used systemic treatment approaches in advanced biliary tract cancer, integrating first-line chemo-immunotherapy, subsequent cytotoxic regimens, and biomarker-directed targeted therapies based on molecular profiling. Clinical trial enrollment should be considered at all stages of treatment.

A phase II trial evaluated FOLFIRINOX in 40 patients who progressed on gemcitabine/cisplatin, using standard and modified dosages (59). mPFS and OS were 6.2 and 10.7 months, respectively, with common grade 3–4 adverse events including neutropenia, diarrhea, nausea, vomiting, and mucositis. Other regimens, such as irinotecan monotherapy, XELIRI (irinotecan and capecitabine), gemcitabine monotherapy, and 5-FU with doxorubicin and mitomycin-C, have been reported (60–65). However, these studies are small and retrospective, lacking sufficient evidence to impact clinical practice. In real-world practice, treatment sequencing following first-line chemo-immunotherapy is often individualized and influenced by performance status, comorbidities, and access to molecular testing. Early comprehensive genomic profiling is increasingly favored to identify actionable alterations before clinical deterioration, although testing may also be performed at progression when tissue is limited at diagnosis. For patients with preserved performance status, fluoropyrimidine-based chemotherapy (most commonly FOLFOX) remains the most widely used second-line option in the absence of a targetable alteration. In older patients, those with impaired renal function, or those who are cisplatin-ineligible, treatment decisions frequently diverge from trial-defined pathways and prioritize tolerability and symptom control. Across all lines of therapy, enrollment in clinical trials remains strongly encouraged whenever available. To facilitate clinical interpretation of the evolving treatment landscape, **Figure 2** provides a schematic overview of contemporary systemic treatment approaches in advanced biliary tract cancer, integrating chemo-immunotherapy, subsequent cytotoxic options, and biomarker-informed targeted therapies.

## 3 Targeted therapies

### 3.1 An overview of the molecular profile of biliary tract cancer

As has been seen in the above, conventional cytotoxic therapies for advanced BTC offer limited survival benefit. Advances in molecular biology of BTC identified a range of genomic alterations, occurring in up to 50% of patients (66). Several potentially targetable mutations were identified that include ARID1A, BAP1, BRAFV600E, BRCA1/2, CDH1, CDKN2A/B, ERBB2/HER2, FGFR2 fusions, IDH1/2, and KRAS (**Table 2**). Interestingly, these mutations vary by biliary tract anatomic subsite. Intrahepatic BTC shows higher frequencies of BAP1 (15–20%), FGFR2 fusions (up to 16%), ARID1A (up to 23%), MSI-H/dMMR (up to 18.2%), and IDH1/2 alterations (67–69). In contrast, extrahepatic BTC and gallbladder tumors are enriched in ERBB2/HER2 (up to 20%), SMAD4 (up to 10.7%), and TP53 (up to 68%), with complete absence of FGFR2 fusions and BAP1 mutations, and lower rates of IDH1/2 (4.7%) and ARID1A (up to 14%) (67, 68, 70–74). Mutations like CDKN2A/B, KRAS, and PI3K are found in both intrahepatic and extrahepatic BTC. Importantly, the population-level clinical impact of individual molecular targets varies substantially based on both prevalence and magnitude of therapeutic benefit. Alterations such as FGFR2 fusions in intrahepatic cholangiocarcinoma and HER2 amplification in extrahepatic disease account for a larger proportion of patients and therefore have broader clinical applicability, whereas alterations such as MSI-H/dMMR, BRAFV600E, or NTRK fusions are less frequent but often associated with deep and durable responses to targeted or immune-based therapies. These distinctions are important when interpreting the overall impact of biomarker-driven strategies in BTC. Understanding these molecular alterations led to the development of targeted therapies, some approved by the FDA, which are discussed in this section (**Table 2**).

**Table 2:** Reported frequency of mutations and genetic alterations based on the anatomic location of cholangiocarcinoma (CCA), with corresponding FDA-approved targeted therapies.

Alteration	eCCA (%)	iCCA (%)	Targetable? (Y/N)	Approved therapy	Year of approval	Major AEs	Comments
KRAS	36.7–46	7-54	N	–	–	–	–
TP53	18-68	18-27	N	–	–	–	–
CDKN2A/B	9-28	9-27	N	–	–	–	–
ARID1A	14	18-23	N	–	–	–	–
HER2	1.3-20	5.8	Y	*Pertuzumab + Trastuzumab*	–	Elevated aminotransferase	ORR: 23%; DOR: 10.8 mo.; m-PFS: 4.0 mo.
				*Zanidatamab*	2024 (FDA accelerated approval)	Diarrhea, infusion-related reactions, nausea, fatigue; predominantly grade 1–2; low rates of grade ≥3 toxicity	Demonstrated durable responses in HERIZON-BTC-01
BAP1	–	15-20	N			–	–
IDH1/2	4.7	13-30	Y	*Ivosidenib*	2021	Ascites, anemia	–
BRAF^V600E^	–	1.5	Y	*Trametinib + dabrafenib*	2022	Leucopenia, neutropenia, pyrexia, fatigue, elevated GGT	Tumor-agnostic approval
BRCA1	2	0.4	N	–	–	–	–
BRCA2	2.5	2.8	N	–	–	–	–
CDH1	–	11.8	N	–	–	–	–
*FGFR2*	0	10-16	Y	*Pemigatinib*	2020	Hyperphosphatemia, stomatitis, arthralgia, ocular toxicities	–
				*Futibatinib*	2022		Distribution cessation/withdrawal by manufacturer
PI3K	5	7	N	–	–	–	–
SMAD4	10.7	–	N	–	–	–	–
MSI-H/dMMR	4	4.7-18.2	Y	*Pembrolizumab*	2020	Diarrhea/colitis, pancreatitis, fatigue, anemia	Tumor-agnostic approval
				*Dostarlimab*	2021		
NTRK fusion	2-3		Y	*Larotrectinib*	2018	Fatigue, weight gain, arthralgia, anemia	–
				*Entrectinib*	2019		
RET fusions	unknown		Y	*Selpercatinib*	2022	Hypertension, elevated aminotransferase	Tumor-agnostic approval

eCCA, extrahepatic CCA; iCCA, intrahepatic CCA; KRAS, Kirsten rat sarcoma viral oncogene homolog; TP53, tumor protein 53; CDKN2A/B, Cyclin-Dependent Kinase N 2A/B; ARID1A, AT-rich interaction domain 1A; HER2, Human epidermal Growth Factor receptor; BAP-1, BRCA1-associated protein 1; IDH1/2, Isocitrate dehydrogenase; BRAF, V-Raf Murine Sarcoma Viral Oncogene Homolog B; BRCA, breast cancer antigen; CDH1, cadherin-1; FGFR, fibroblast growth factor receptor; PI3K, phosphoinositide-3-kinase; SMAD4, mothers against decapentaplegic homolog 4; MSI-H, microsatellite instability-high; dMMR, deficient mismatch repair; NTRK, Neurotrophic tyrosine receptor kinase; RET, Re-arranged during transfection; Y, Yes; N, No; GGT, Gamma-glutamyl transferase.

#### 3.1.1 Biomarker limitations

While biomarker testing in BTC holds promise for personalized targeted therapy, its real-life application faces significant challenges, particularly with tissue acquisition. Advanced disease often precludes surgical resection, and diagnostic needle biopsies are limited by insufficient tumor cells for molecular characterization, as well as inadequate sample quality (75). Failure rates for tissue-biopsy profiling in metastatic BTCs are reported to reach 26.8% (76). Given the increasing therapeutic relevance of actionable genomic alterations in BTC, obtaining adequate tissue for molecular profiling is essential whenever feasible. Core needle biopsy is generally preferred over fine-needle aspiration alone, as limited cytologic samples may be insufficient for comprehensive next-generation sequencing and biomarker assessment.

Liquid biopsy assays have been proposed as an alternative and complement to tissue-based sampling for BTC and are now supported by a growing body of prospective data. Recent studies and reviews demonstrate acceptable concordance between ctDNA and tissue NGS for identifying actionable alterations in advanced BTC and support ctDNA as a feasible platform for genomic profiling when tissue is inadequate or unobtainable. At the same time, important limitations remain, including variable ctDNA shedding, lower sensitivity for certain genomic events (particularly low-VAF mutations and some gene fusions), and the need for standardized assay platforms and reporting frameworks. Emerging data also suggest that ctDNA dynamics may have prognostic value—higher baseline cfDNA/ctDNA levels and persistent or rising ctDNA during therapy have been associated with worse PFS/OS or earlier recurrence after resection in BTC (77–80).

#### 3.1.2 Fibroblast growth factor 2 fusions

FGFR2 fusions, observed in 10–20% of iCCA cases, activate downstream signaling pathways that drive tumorigenesis, promoting cell proliferation, survival, and angiogenesis (81–83). These fusions are more common in younger women and are a defining molecular feature of iCCA (2). Multiple FGFR inhibitors have been developed, transforming the treatment landscape. Pemigatinib, the first FDA-approved FGFR-targeted therapy, demonstrated an ORR of 35.5%, mPFS of 6.9 months, and mOS of 21.1 months in the FIGHT-202 trial for previously treated FGFR2 fusion-positive iCCA and is currently being studied in the first-line setting (FIGHT-302) (84, 85). Futibatinib, a covalent FGFR1–4 inhibitor, showed superior efficacy in the FOENIX-CCA2 trial, achieving an ORR of 42%, mPFS of 9.0 months, and mOS of 21.7 months. Unlike its predecessors, futibatinib targets resistance mutations commonly associated with ATP-competitive inhibitors, reducing drug-resistant clones (86–88). Other inhibitors, such as derazantinib and erdafitinib, have shown promise in early studies (89, 90). Despite these advancements, challenges remain, including understanding the clinical implications of more than 150 identified FGFR2 fusion partners and co-occurring mutations. Initial genomic analyses suggest limited impact of these factors on treatment efficacy, but larger studies are needed to confirm these findings (91). FGFR inhibitors are now integral in managing FGFR2 fusion-positive iCCA, with ongoing trials evaluating their potential in the first line setting.

### 3.2 Isocitrate dehydrogenase isoenzyme mutations

Mutations in IDH1/2 are among the most common genomic alterations in iCCA, with a prevalence of 13% to 36% (92–94). These mutations, often associated with infectious etiologies of the biliary tree (95), result in the production of the oncometabolite 2-hydroxyglutarate (2-HG), which promotes tumorigenesis by inhibiting cellular differentiation through histone modification (96–98).

Ivosidenib, a first-in-class oral IDH1 inhibitor, was initially studied in a phase I trial that included 73 patients with IDH1-mutated CCA, reporting an ORR of 5%, mPFS of 3.8 months, and mOS of 13.8 months, with 56% achieving stable disease (99). Responders showed reductions in circulating 2-HG levels, and the drug was well tolerated. In the phase III ClariDHy trial, ivosidenib (500 mg daily) was compared to placebo in 185 patients with IDH1-mutated iCCA in the second- and third-line settings. The trial demonstrated significantly improved outcomes with ivosidenib, including an ORR of 2%, mPFS of 2.7 months, and mOS of 10.3 months, compared to 5.1 months for placebo (94, 100). These results led to FDA approval of ivosidenib for previously treated patients with unresectable or metastatic IDH1-mutated CCA (101).

Other IDH inhibitors are under investigation in clinical trials (e.g., NCT02481154, NCT02746081), and preclinical models suggest that 2-HG may increase tumor sensitivity to Poly-adenosine diphosphate-ribose polymerase (PARP) inhibitors, prompting ongoing studies evaluating this combination in IDH1/2-mutated CCA (NCT03212274; NCT03878095).

### 3.3 *BRAF*^V600E^ mutations

The Raf murine sarcoma viral oncogene homology B (BRAF)V600E mutation, a substitution of valine (V) with glutamic acid (E) at amino acid 600, leads to constitutive activation of the BRAF protein, driving the mitogen-activated protein kinase (MAPK)/extracellular signal-regulated kinase (ERK) pathway and promoting oncogenesis. This mutation is more commonly observed in iCCA compared to eCCA or GBC, with prevalence rates in iCCA varying between 1% and 22% in case series and a reported 1.5% for BRAFV600E specifically. Variability in prevalence underscores the heterogeneity of these tumors (102–109).

Dabrafenib, a competitive RAF inhibitor, and trametinib, a selective MEK1/2 inhibitor, are commonly combined to target BRAFV600E-mutated tumors. Dabrafenib reduces MEK and ERK phosphorylation, leading to cell cycle arrest and caspase activation, while trametinib prevents resistance to dabrafenib by selectively inhibiting MEK1/2 (106, 107). In the phase 2 ROAR trial, this combination showed an ORR of 51%, with a mOS of 14 months and PFS of 9 months in 43 patients with CCA (106). Similarly, the NCI-MATCH trial reported an ORR of 38%, DOR of 25.1 months, mOS of 28.6 months, and mPFS of 11.4 months, including PRs in 3 of 4 CCA patients (107). These results supported FDA approval of the combination for BRAFV600E-mutated, unresectable, or metastatic solid tumors in patients aged >6 years who progressed on prior therapy (108).

Binimetinib, another MEK1/2 inhibitor, has been studied in combination with capecitabine in 34 gemcitabine-refractory CCA patients, achieving an ORR of 20.6% (all PRs) and a mOS of 7.8 months. Patients with RAS/RAF/MEK/ERK pathway mutations exhibited improved outcomes, with an ORR of 40%, PFS of 5.4 months, and OS of 10.8 months compared to wild-type patients (109).

### 3.4 ERBB2/HER-2 amplifications

Amplifications in the erythroblastic oncogene B-2, (ERBB2) encoding HER-2 (human epidermal growth factor receptor 2), are most commonly reported in eCCA, including GBC, with a prevalence of up to 20% compared to 6% in iCCA (73, 74). HER-2 activation triggers downstream signaling pathways involved in tumor growth, and its amplification promotes tumorigenesis (74, 110, 111). In the phase 2 MyPathway trial, 39 HER-2-positive metastatic CCA patients received pertuzumab and trastuzumab, resulting in an ORR of 23%, a mDOR of 10.8 months, and mPFS of 4.0 months (112).

Pan-HER kinase inhibitors are under evaluation. The SUMMIT basket trial (NCT01953926) showed an ORR of 10% among 20 BTC patients treated with neratinib (113, 114). Other inhibitors, such as lapatinib and afatinib, have been studied in BTC without favorable results. T-Dxd demonstrated promise in HER-2-positive advanced solid tumors, including CCA, with a phase 1 trial showing an ORR of 28.3% and mPFS of 7.2 months (115). The HERB phase 2 trial evaluated 32 BTC patients, reporting an ORR of 36.4%, mPFS of 4.4 months, and mOS of 7.1 months for HER-2-positive tumors. In comparison, HER-2-low patients had an ORR of 12.5%, mPFS of 4.2 months, and mOS of 8.9 months (116).

Zanidatamab is a bispecific antibody that targets two distinct extracellular domains of the HER2 receptor, leading to receptor clustering, internalization, and degradation, thereby inhibiting tumor growth. In the phase 2b HERIZON-BTC-01 study, previously treated, unresectable or metastatic HER2-positive (IHC3+) BTC, zanidatamab achieved an ORR of ~41% with a median duration of response of ~13 months and a 9-month OS rate close to 70% (117).

Beyond monoclonal antibodies, small-molecule HER2 TKIs are also showing activity in BTC. In the phase II basket study SGNTUC-019, tucatinib plus trastuzumab in previously treated HER2-positive metastatic BTC produced a confirmed ORR of 46.7%, disease-control rate of 76.7%, median PFS 5.5 months and an estimated 12-month OS rate of 53.6%, with a tolerable safety profile (118).

Ado-trastuzumab emtansine (T-DM1) is an antibody-drug conjugate that combines trastuzumab, a monoclonal antibody targeting the HER2 receptor, with the cytotoxic agent DM1. This design allows for targeted delivery of DM1 to HER2-positive cancer cells, leading to cell death while minimizing damage to normal tissues. In a phase 2 trial involving patients with HER2-positive biliary tract adenocarcinoma, T-DM1 demonstrated a confirmed ORR of 12.5%, with a mPFS of 3.1 months and a mOS of 7.1 months (119).

### 3.5 High microsatellite instability and mismatch repair deficiency

Deficiency in DNA mismatch repair (dMMR) and high microsatellite instability (MSI-H) lead to the accumulation of mutations due to higher rates of unrepaired DNA replication errors. These mutations manifest as neoantigens on cancer cells, attracting CD8+ T-cell infiltration in the tumor microenvironment (120). This mechanism underpins the success of immune checkpoint inhibitors, particularly in highly mutagenic malignancies (121). In BTCs, MSI-H/dMMR is more prevalent in iCCA (4.7–18.2%) compared to eCCA (4%) (70, 122).

Two immune checkpoint inhibitors, pembrolizumab and dostarlimab, are FDA-approved for MSI-H/dMMR solid tumors, including BTCs. Pembrolizumab was approved based on the KEYNOTE-158 trial and a phase 2 study by Le et al., for unresectable or metastatic MSI-H/dMMR tumors progressing after prior treatment (123). Dostarlimab, supported by the phase 1 GARNET study, is approved for MSI-H/dMMR recurrent or advanced solid tumors progressing after prior therapy (124). Both trials included BTC patients, with further details covered in the immunotherapy section, alongside other immune checkpoint inhibitors and combination approaches.

### 3.6 Other targets and targeted therapies

Several other molecular targets and associated therapies have been explored in BTCs.

#### 3.6.1 cMET

cMET amplification and overexpression, observed in 2–8% of BTCs, are linked to tumor invasion, angiogenesis, and poor prognosis. Elevated cMET expression is reported in 15% of eCCA and 12% of iCCA (125, 126). Cabozantinib, a multikinase inhibitor targeting MET, showed limited activity and significant toxicity in a phase II trial of 19 CCA patients (127).

#### 3.6.2 PI3K/AKT/mTOR pathway

Aberrations in this pathway occur in up to 25% of iCCA, 40% of eCCA, and 4–16% of GBC (128). While inhibitors targeting PI3K, AKT, and mTOR have been studied in first- and second-line settings, they have shown minimal efficacy (129–131). Combination strategies with chemotherapy or other targeted therapies are being investigated.

#### 3.6.3 PARP and PARP inhibitors

DNA damage repair gene alterations, including BRCA1/2 mutations, occur in up to 63% of BTCs (132–134). Although retrospective data on PARPi in BRCA-mutant BTCs are inconclusive, no clinical trials have evaluated their efficacy in first- or second-line settings (135). Recently, however, a phase II study evaluated the efficacy of niraparib, a PARP inhibitor, in patients with BRCA-mutated unresectable or recurrent biliary tract, pancreatic, and other gastrointestinal cancers. The study found that niraparib demonstrated promising antitumor activity in this patient population (136).

#### 3.6.4 Neurotrophic tyrosine receptor kinase gene fusions and NTRK inhibitors

NTRK1/2/3 gene fusions, activating PI3K and MAPK pathways, are found in 2–3% of CCA cases (137–139). Larotrectinib, a pan-NTRK inhibitor, achieved an ORR of 80% and a 1-year PFS of 55% in a phase II trial, including 2 CCA patients (140, 141). Entrectinib demonstrated an ORR of 57%, mPFS of 11.2 months, and mOS of 21 months in the phase II STARTRK-2 study, leading to FDA approval for NTRK fusion-positive solid tumors (142).

#### 3.6.5 RET (rearranged during transfection) gene fusion/re-arrangement and RET inhibitors

RET fusions drive oncogenesis through downstream pathway activation. Prevalence in BTCs is unclear (143). In the phase I/II ARROW trial, pralsetinib achieved an ORR of 57%, mPFS of 7.4 months, and mOS of 13.6 months in RET fusion-positive solid tumors, including 3 CCA patients (144, 145). Selpercatinib, evaluated in the LIBRETTO-001 trial, showed an ORR of 43.9%, mPFS of 13.2 months, and mOS of 18 months, with 2 CCA patients included (146, 147). Further studies are needed to clarify the role of RET inhibitors in BTCs.

## 4 Immunotherapy (**Table 1**)

### 4.1 Immune checkpoint inhibitors monotherapy and combination

Following the success of ICIs in gastrointestinal tumors, including hepatocellular carcinoma (148), their use in BTCs has been extensively evaluated.

#### 4.1.1 ICI monotherapy

Pembrolizumab was studied in the phase 1b KEYNOTE-028 trial with 24 PD-L1-positive BTC patients, showing a 17% PR and 17% SD rate (149). In KEYNOTE-158, 104 unselected pre-treated BTC patients had an ORR of 5.8%, with mOS and mPFS of 7.4 and 2.0 months, respectively. Retrospective analysis revealed that PD-L1 positivity (58.7%) was associated with a higher ORR (6.6% *vs*. 2.9%) but not superior survival outcomes (150). Nivolumab demonstrated promise in phase 1 and 2 trials, with a phase 2 study reporting an ORR of 22%, DCR of 59%, mPFS of 3.7 months, and mOS of 14.2 months in 46 advanced refractory BTC patients. PD-L1 positivity correlated with longer PFS but not OS (151, 152). Durvalumab, tested as monotherapy and combined with tremelimumab in a phase 1 trial of pre-treated Asian BTC patients, achieved mOS of 8.1 months and 10.1 months, respectively (153).

#### 4.1.2 ICI combination therapy

The combination of nivolumab and ipilimumab was evaluated in the phase 2 CA209–538 trial in 39 BTC patients (16 iCCA, 10 eCCA, 13 GBC). This combination achieved an ORR of 23%, DCR of 44%, mPFS of 2.9 months, and mOS of 5.7 months (154).

#### 4.1.3 ICIs and chemotherapy combination

The potential synergy of chemotherapy and immunotherapy was explored extensively in BTC, with gemcitabine/cisplatin (GEMCIS) as the most common chemotherapy backbone. In a phase 2 study of 32 BTC patients, nivolumab combined with GEMCIS achieved an ORR of 61.9% in treatment-naïve patients and 33% in pre-treated patients, with no significant differences in PFS or OS (155). Toripalimab, an anti-PD1 inhibitor, was evaluated with S-1 and gemcitabine in a phase 2 trial of 48 treatment-naïve patients, achieving a mPFS of 7 months, mOS of 16 months, and an ORR of 27.1% with a DCR of 87.5% (156). Camrelizumab, combined with oxaliplatin-based regimens GEMOX and FOLFOX, demonstrated ORRs of 16.3–54%, mPFS of 5.3–6.1 months, and mOS of 11.8–12.4 months, with better responses in PD-L1-positive patients (ORR 80% *vs*. 53.8%) (157, 158).

Durvalumab-based and pembrolizumab-based chemo-immunotherapy regimens have demonstrated broadly comparable survival benefits, establishing PD-1/PD-L1 blockade plus gemcitabine/cisplatin as a class effect rather than a drug-specific advantage. In a phase 2 study of 121 chemotherapy-naïve BTC patients, GEMCIS plus durvalumab achieved an ORR of 73.4%, mPFS of 11 months, and mOS of 18.1 months. Comparable outcomes were seen with the addition of tremelimumab (159). The subsequent TOPAZ-1 phase 3 trial randomized 685 treatment-naïve patients with metastatic or recurrent BTC to GEMCIS plus durvalumab or placebo. Durvalumab improved ORR (26.7% *vs*. 18.7%), 2-year OS rates (24.9% *vs*. 10.4%), and reduced hazard ratios for OS (0.80; p=0.021) and PFS (0.75; p=0.001) (160). Based on these results, the FDA approved GEMCIS plus durvalumab for advanced BTC, establishing it as a new standard first-line therapy and a pivotal change in the treatment landscape since the ABC-02 trial (161).

The phase 3 Keynote-966 trial randomized 1,069 untreated BTC patients to receive gemcitabine/cisplatin with either pembrolizumab or placebo. Pembrolizumab significantly improved OS (12.7 *vs*. 10.9 months; p=0.0034) without increasing toxicity, establishing it as a new first-line option for BTC (162).

The phase 2 IMbrave151 trial evaluated the addition of bevacizumab to atezolizumab, gemcitabine, and cisplatin in 162 untreated BTC patients. PFS was slightly higher in the bevacizumab arm (8.4 *vs*. 7.9 months), but the difference was not clinically meaningful (163).

Racial differences may influence ICI efficacy in BTC. Cross-trial comparisons indicate higher response rates in Asian patients, as observed in studies like TOPAZ-1, where subgroup analysis showed greater benefit for Asian patients (150, 151, 153, 160). Geographic and molecular heterogeneity in BTC, including differences in tumor biology and risk factors, highlight the importance of considering these factors in trial design (164, 165). The neutral results of IMbrave151 suggest that the addition of anti-angiogenic therapy to chemo-immunotherapy may not confer incremental benefit in unselected BTC populations. Future studies may need to incorporate biomarker selection or alternative combination strategies to better define subsets most likely to benefit.

## 5 Novel agents and major ongoing trials

The BTC treatment landscape is rapidly advancing with the development of targeted therapies and ICIs. Multiple ongoing trials are investigating novel agents and combinations.

In targeted therapies, newer FGFR2 inhibitors are being developed to address both primary and acquired resistance to first-generation agents. Pemigatinib and futibatinib are now standard options for previously treated FGFR2 fusion-positive iCCA, while infigratinib has been withdrawn from the BTC market and repurposed for non-oncologic indications (88). A key emerging concept is sequential FGFR inhibition: resistance to one FGFR TKI is frequently mediated by heterogeneous secondary FGFR2 kinase-domain mutations, and next-generation inhibitors such as tinengotinib have shown activity in patients who have progressed on prior FGFR inhibitors. Phase I/II data suggest meaningful disease control and mPFS of ~5–6 months in heavily pre-treated FGFR-altered CCA, and the ongoing phase III FIRST-308 trial is specifically evaluating tinengotinib versus investigator’s-choice chemotherapy (FOLFOX or FOLFIRI) in patients previously treated with both chemotherapy and a first-generation FGFR inhibitor (166, 167).

The pembrolizumab plus lenvatinib combination, studied in the phase 2 LEAP-005 trial with 31 pre-treated advanced BTC patients, showed encouraging results, prompting expansion to 100 patients. Final analysis is pending (168). Other combinations under investigation include atezolizumab plus cobimetinib (169), and toripalimab with lenvatinib (NCT04211168) or lenvatinib plus GEMOX (170).

In the first-line setting, several chemo-IO combinations have been explored beyond durvalumab. The phase II EORTC-1607 trial evaluated pembrolizumab plus gemcitabine/cisplatin in unresectable or metastatic BTC (171). Recently reported data showed ORR >40%, median PFS >8 months and median OS ~13–14 months, broadly comparable to other chemo-IO regimens but without clear superiority over TOPAZ-1 or KEYNOTE-966. These results support the general principle that adding PD-1/PD-L1 blockade to gemcitabine/cisplatin improves outcomes versus chemotherapy alone, but do not establish pembrolizumab-gemcitabine/cisplatin as a distinct standard of care.

Nivolumab-based regimens, including with S-1 plus gemcitabine (NCT04172402) and nal-irinotecan plus 5-FU/leucovorin (NCT03785873), are also under evaluation (172).

Lastly, several trials investigating vaccine-based therapies in patients with BTCs have been initiated, at various stages: in the first line setting and in patients with Globo H-positive advanced BTC who did not progress after gemcitabine and cisplatin, a phase II clinical trial is investigating the safety and efficacy of OBI-833/OBI-821 as a maintenance therapy in combination with ICI (NCT06490198). OBI-833/OBI-821 is a novel cancer vaccine targeting a tumor-associated carbohydrate antigen, Globo H, with proven safety and efficacy (173). Another phase 1 clinical trial is studying the safety and immune response of URLC10, a peptide-based vaccine therapy, in combination with gemcitabine, in patients with unresectable or recurrent BTC (NCT00624182). Similarly, a pilot trial is evaluating the safety and efficacy of mBTCvax, a personalized mutant peptide vaccine targeting driver oncogenes, in combination with durvalumab and tremelimumab following front-line treatment in patients with advanced stage BTC (NCT06564623). **Table 3** provides an overview of ongoing and recently closed trials in advanced BTC.

**Table 3:** Ongoing phase III clinical trials in patients with advanced/unresectable or metastatic BTC.

NCT	Title	Arm 1	Arm 2	Status	Location	Comments
05924880	A Phase 3b, Open-label, Multi-center Study on Durvalumab in Combination with Gemcitabine-based chemotherapy as 1L Treatment for the Chinese Patients with Unresectable BTC	Durvalumab + Gemcitabine-based Chemotherapy	–	Recruiting	China	Single Arm
00939848	Cediranib versus Placebo Plus Cisplatin/Gemcitabine Chemotherapy for Patients with Advanced BTC	Cediranib + Chemotherapy	Placebo + Chemotherapy	Completed/Results pending	UK	
03478488	KN035-BTC: Programmed Death Ligand Combined with Chemotherapy for patients with BTC	KN035 + Gemcitabine-based chemotherapy	Gemcitabine/ Oxaliplatin	Recruiting	China	Open-label
05771480	TOURMALINE: Durvalumab With Chemotherapy as First-line Treatment in Patients with Advanced BTC	Durvalumab + gemcitabine-based chemotherapy	–	Recruiting	US, Europe, Japan, Korea	Single Arm; 7 different chemo regimens
05429697	Study of SMT-NK Inj. Plus pembrolizumab vs. Pembrolizumab monotherapy in Patients with Advanced BTC	SMT-NK + Pembrolizumab	Pembrolizumab monotherapy	Recruiting	Korea	
03093870	Varlitinib in Combination with Capecitabine for Advanced/Metastatic BTC as Second Line Systemic Therapy	Varlitinib + Capecitabine	Placebo + Capecitabine	Completed with results	US, Australia, China, Europe, Hong Kong, Korea, Japan, Singapore	
05065957	Study of Combination Therapy of D07001-Softgel Capsules and Xeloda/TS-1 in Subjects with Advanced BTC, after Gemcitabine/Cisplatin-based treatment failure	D07001 + Xeloda (or TS-1)	mFOLFOX	Recruiting	Taiwan	
05506943	COMPANION-002: A study of CTX-009 in Combination with Placlitaxel in Adult Patients with Unresectable Advanced, Metastatic or recurrent BTC	CTX-009 + Paclitaxel	Paclitaxel	Recruiting	US	
01149122	Gemcitabine/Oxaliplatin (GEMOX) with or without Erlotinib in Advanced BTC	GEMOX + Erlotinib	GEMOX alone	Completed/Results pending	Korea	Open label
06490198	Phase 2 Study: OBI-833/OBI-821 Maintenance for Globo H+ Advanced BTC after Gemcitabine/Cisplatin	OBI-833/OBI-821 + ICI	–	Not yet recruiting	Taiwan	Single-arm, open-label
00624182	Gemcitabine with Peptide Vaccine Therapy in Treating Patients with Bile Duct Cancer	URLC10 + Gemcitabine	–	Suspended	Japan	Single-arm, open-label
06564623	Targeting Driver Oncogenes With a Peptide Vaccine Plus Durvalumab and Tremelimumab for Patients with BTC	mBTCvax + Durvalamab + Tremelimumab	–	Not yet recruiting	US	Single-arm, open-label

As published online at www.clinicaltrials.gov (last accessed 10/12/2023). The table only includes phase III clinical trials that are active/actively recruiting or recently closed trials (within 2 years) with or without published results. BTC: biliary tract cancer; 1L: first-line; US: United States; UK: United Kingdom; chemo: chemotherapy; mFOLOFX: modified FOLFOX (Folinic acid, leucovorin, fluorouracil, and oxaliplatin).

## 6 Conclusion and future direction

Despite the availability of multiple FDA-approved therapies, the prognosis for BTC remains poor, primarily because most patients present with unresectable or metastatic disease. Chemo-immunotherapy has become a cornerstone of first-line treatment: TOPAZ-1 established gemcitabine/cisplatin plus durvalumab as a new standard of care, and KEYNOTE-966 confirmed a survival benefit with gemcitabine/cisplatin plus pembrolizumab versus chemotherapy alone. In later lines, FGFR2 and IDH1 inhibitors, HER2-directed antibodies (including zanidatamab), and HER2-TKI combinations (such as tucatinib plus trastuzumab) have expanded options for molecularly selected patients, while ongoing trials of next-generation FGFR inhibitors (e.g., tinengotinib in FIRST-308) are testing strategies for sequential targeted therapy.

Molecular profiling has become a critical component of BTC management, particularly for advanced cases, enabling the identification of actionable mutations and guiding personalized therapy. Ongoing clinical trials are exploring novel combinations of immunotherapy, chemotherapy, and targeted agents to improve efficacy while minimizing toxicity. These developments highlight a rapidly evolving landscape, bringing the promise of better outcomes for patients with this challenging malignancy. Patients with BTC must always be considered for inclusion in ongoing clinical trials.
